# A continuous model of physiological prion aggregation suggests a role for Orb2 in gating long-term synaptic information

**DOI:** 10.1098/rsos.180336

**Published:** 2018-12-12

**Authors:** Michele Sanguanini, Antonino Cattaneo

**Affiliations:** Scuola Normale Superiore, Piazza dei Cavalieri, 7, 56126 Pisa, Italy

**Keywords:** amyloid, physiological prion, Orb2, long-term memory, synaptic plasticity

## Abstract

The regulation of mRNA translation at the level of the synapse is believed to be fundamental in memory and learning at the cellular level. The family of cytoplasmic polyadenylation element binding (CPEB) proteins emerged as an important RNA-binding protein family during development and in adult neurons. *Drosophila* Orb2 (homologue of mouse CPEB3 protein and of the neural isoform of *Aplysia* CPEB) has been found to be involved in the translation of plasticity-dependent mRNAs and has been associated with long-term memory. Orb2 protein presents two main isoforms, Orb2A and Orb2B, which form an activity-induced amyloid-like functional aggregate, thought to be the translation-inducing state of the RNA-binding protein. Here we present a first two-states continuous differential model for Orb2A–Orb2B aggregation. This model provides new working hypotheses for studying the role of prion-like CPEB proteins in long-term synaptic plasticity. Moreover, this model can be used as a first step to integrate translation- and protein aggregation-dependent phenomena in synaptic facilitation rules.

## 1. Background

Different and complex cellular and molecular aspects underlie information learning and memory, even in simple invertebrate models such as *Aplysia californica* or *Drosophila melanogaster* [1,2]. A commonly used framework to model memory and learning phenomena is founded on the Hebb principle, which states that the synaptic connection between two neurons is facilitated if the neuronal pre-synaptic and post-synaptic activity correlate [3] consistently. A direct experimental validation of Hebb principle is provided by the phenomenon of *Spike Time-Dependent Plasticity* (STDP) [4]. In general, synaptic learning rules should involve the activation of long-lasting mechanisms that are local in nature (to ensure local synaptic-specificity of the plasticity event). A commonly used synaptic rule is derived from STDP for modelling neuronal network simulations [5,6] and increases or decreases the synaptic efficacy according to the time difference in the onset of the pre- and the post-synaptic activity. However, this rule usually does not take into account molecular processes which are fundamental in establishing long-term memory, such as the local translation at the synapse [7,8].

The CPEB proteins are translational regulators of specific mRNA targets which carry the conserved CPE regulatory sequence [9–11]. All CPEB proteins are present in two states, a translation-inducing state and a repressor one [12–17], and act as binary switches of local synaptic translation. *Drosophila* Orb2 protein, a member of the CPEB family, shows an intrinsically disordered N-terminus [10,13,18,19], which triggers the transition to a translation-permissive state through the induction of prion-like templating and amyloid aggregation. These amyloid-like aggregates are self-sustaining, self-templating [20] and functionally correlated with Long-Term Memory (LTM) [15]. Interestingly, the mouse isoform CPEB3 [16,17] shows a similar mechanism of activation upon protein aggregation, suggesting a role for functional amyloid-like aggregation in mammals.

Some of the mRNAs that are transcribed from the *orb2* gene produce two protein variants [10], the isoform A (Orb2A) and B (Orb2B) [10,13,18], that have complementary roles in LTM-related tasks [18]. Orb2B is the most abundant isoform in the cell, is widely diffused and shows no, or low, aggregation propensity; Orb2A is expressed at low levels, accumulates after the induction of LTM protocols and swiftly forms aggregates with biochemical features (detergent- and protease-resistance) that are typical of amyloid-like structures [13]. It has been shown that Orb2A mediates the local aggregation of Orb2B after synaptic stimulation, via the formation of a heteromeric Orb2 aggregate [13,18] which regulates the target mRNA translation [20].

The aim of this paper is to introduce and characterize the properties of a simple model of Orb2 aggregation, in order to gain insight into the properties of CPEB prion-like aggregation and produce hypotheses that can be tested experimentally. We also propose this model, which implicitly and explicitly involves the local protein translation at the synapse, as the first step to include in synaptic facilitation rules facilitation phenomena determined by protein aggregation-dependent plasticity (PADP) mechanisms.

## 2. Results

Little is known about the kinetics of Orb2 activity-dependent activation and its precise mechanism of aggregation *in vivo*. At resting state, there are undetectable levels of Orb2A in the synapse [13] due to the intrinsic instability of the Orb2A protein, which is stabilized through phosphorylation upon synaptic activity [21]. In these conditions, it is likely that the accumulated Orb2A forms an aggregation seed [22] that induces the hetero-oligomerization of the soluble isoform Orb2B [15,18]. As a confirmation of this hypothesis, it has also been shown that the Orb2A isoform is necessary during memory acquisition and that the Orb2B isoform is required during memory consolidation [23]. Also, it seems that a neuromodulatory dopaminergic stimulation is necessary for both memory formation steps [23,24]. Moreover, Orb2 aggregates are self-sustaining and self-limiting, such as ApCPEB amyloids [13], which could imply the presence of a tight network of regulatory pathways at the synapse.

**Figure 1.**
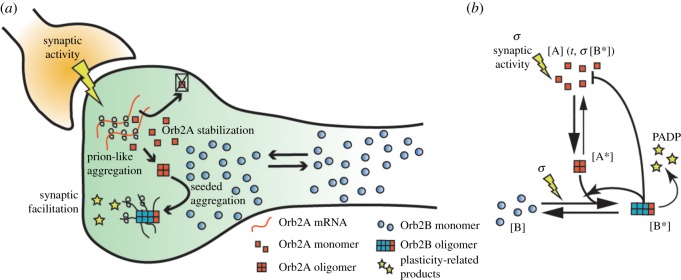
Orb2 prion-like physiological amyloid aggregation. (*a*) Schematic representation and (*b*) reaction scheme of the prion-like aggregation mechanism of Orb2 presented in the model: the equilibrium between monomeric and aggregated Orb2A and Orb2B depends on the synaptic activity and the levels of Orb2 itself. [A], monomeric Orb2A; [A*], aggregated Orb2A; [B], monomeric Orb2B; [B*], aggregated Orb2B; *t*, time; arrow, induction of reaction; bar, inhibition of reaction.

Given the low amount of mechanistic information about Orb2 aggregation [19,25], we decided to address it from a phenomenological point of view, in order to model the general properties of the biological system. We modelled the Orb2 aggregation system using a system of continuous differential equations which follows the reaction scheme of figure 1 (see Methods). The aim of this model is to capture the peculiar features of Orb2 prion-like aggregation and it can be seen as an original extension of a classic model by Tompa and Friedrich on prion protein aggregation [26] that includes three prion protein (PrP) structural isoforms (PrP^C^, PrP^*^ and PrP^A^), with PrP^A^ the self-templating form. The model from Tompa and Friedrich shows that prions have the potentiality to form conformational bistable switches. A chaperon X induces the conversion of PrP^C^ to PrP^*^, that can further convert to PrP^A^ with a slow kinetic. PrP^A^ then catalyses its own production from PrP^*^. Both PrP^*^ and PrP^A^ can be degraded by a protease D, with PrP^*^ having a much higher propensity to degradation. In our model, Orb2A has a function similar to chaperon X, with the fundamental differences that we do not model an Orb2B aggregation-prone intermediate conformational state and that the mature Orb2 aggregate provides a negative feedback on Orb2A production.

### 2.1. Description of the Orb2 aggregation model

In order to model, in a first approximation, the Orb2 aggregation we decided to model four species: monomeric Orb2A (A) and Orb2B (B), and the relative aggregated forms (A^*^ and B^*^). We decided not to model intermediate aggregation-prone monomeric species, as Orb2A has a strong aggregation propensity, and aggregation-defective Orb2B does not determine a loss in LTM consolidation [18]. For the sake of abstraction, we are also considering the aggregate form to be a general state of the proteins, regardless of whether they form many short polymers or few long ones.

To simplify the model, we had to make some important assumptions. We consider the synapse as a punctiform compartment, that is there is no effect of the size of the synapse on the ‘availability’ of the A and B species in the aggregation reaction. This is biologically justified in the case that there is a focal production of Orb2A and Orb2B is locally abundant. We also assume there is no diffusion of the Orb2A protein outside the synapse. There is evidence that Orb2A interacts with lipid membranes [27] and this interaction might restrict the diffusion of this protein species. Moreover, we assume that the steady-state level of the Orb2A protein in the synapse is zero and its accumulation—which is likely to involve multiple mechanisms at both mRNA processing [28] and protein stability levels—depends only on synaptic activity. Experimental evidence suggests that the aggregation propensity of Orb2A makes this isoform a potential burden for cell proteostasis and thus the Orb2A local level is strictly regulated by the cell through a finely tuned translation and degradation equilibrium [21]. This equilibrium is strongly dependent on synaptic activity [21,23]. From the previous three conditions, it comes that the studied variables (A, B, A^*^ and B^*^) are only functions of time and their variation is a total derivative with respect to *t*.

We designed the model according to the hypothesis that the aggregation seed is composed by Orb2A oligomers [18,21,23,25], while the Orb2 mature aggregate is composed by the abundant Orb2B isoform and, once formed, is completely self-sustaining. The formation of the aggregate then would activate the translation of plasticity-related proteins, such as CamKII [23], that are structural and molecular mediators of synaptic facilitation.

### 2.2. Properties of the model and dependency on the characteristics of the stimulus

Orb2 aggregation occurs following the downstream signalling to neuromodulators such as tyramine [15] and dopamine [15,23]. From an experimental point of view, it is possible to induce a strong Orb2 aggregation through the feeding of sucrose and neurotransmitter, with the oligomeric form of Orb2 being evident after 2 h of continuous exposition [15]. A close physiological equivalent to this kind of stimulation is a dopaminergic stimulation with uniform frequency spectrum and a high amplitude that arises during a spaced LTM-related training and can last for hours [29]. For this reason, we used low-frequency, long-lasting stimulation patterns in the form or rectangular functions (see Material and methods) to represent a dopaminergic stimulation with relatively slow neurotransmitter dynamics.

**Figure 2.**
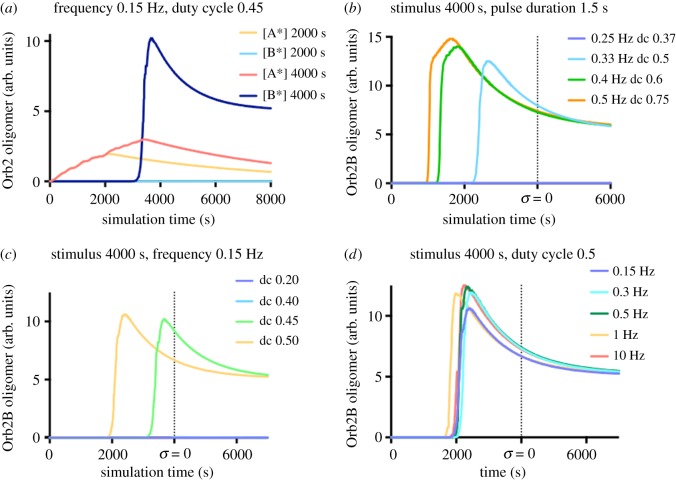
Dynamics of the model in the presence of different stimulation patterns. (*a*) The model of Orb2 aggregation is selective for the length of the stimulation. A relatively short rectangular stimulation (*ν* = 0.15 Hz, dc = 0.45) of 2000 s does not lead to a stable increase in Orb2A levels, nor to the formation of Orb2B aggregates. Increasing the duration of the stimulation (4000 s) leads to a strong increase of Orb2A levels and the formation of a stable, self-sustaining Orb2B aggregate. (*b*) Multiple simulations of the Orb2B aggregation model where the stimulus function is a rectangular (stimulation length 4000 s) wave with varying frequency and duty cycle, in order to get an evenly spaced 1.5 s stimulation. (*c*) The use of rectangular waves with the same frequency (*ν* = 0.15 Hz) and different duty cycles shows a strong duty cycle-dependent effect on Orb2B modelled aggregation dynamics. (*d*) The use of rectangular waves with the same duty cycle (dc = 0.5) and different frequencies shows that the model aggregation dynamics are very similar, and the differences are not frequency-dependent.

Using a rectangular stimulation function with a frequency of 0.15 Hz and a duty cycle of 0.45, we tested equations (4.3), (4.4), (4.5) and (4.6) as shown in Material and methods (from now on called ‘the model’) in order to assess: (i) the sensitivity to the length of the stimulation and (ii) the self-limited growth in presence of a prolonged stimulation. The first property can be proven simulating the previously mentioned stimulation pattern with two intervals: a shorter one, that has not been associated with the production of Orb2 oligomers (2000 s), and a longer time window (4000 s), when Orb2 aggregation starts being evident experimentally [15]. From figure 2*a*, it can be noticed that the shorter stimulation does not manage to trigger the formation of an Orb2A oligomer with seeding ability towards Orb2B.

To answer the second point, we considered simulations where we varied the ‘off’ period of the stimulation function while keeping the ‘on’ period fixed (1.5 s). In order to achieve this pattern of stimulation, we tuned frequency (*ν*) and duty cycle (*dc*) of a rectangular wave (see equation (4.10)). For all simulations, the stimulation ended after 4000 s (see also figure 2*a*). From figure 2*b* it can be seen that there is a threshold of frequency and duty cycle (in our model being between 0.25 and 0.33 Hz, and *dc* between 0.37 and 0.5) which leads to the self-sustaining aggregation. One can see that, after the production of aggregated Orb2, the presence of excess stimulation does not affect the growth of [B^*^].

**Figure 3.**
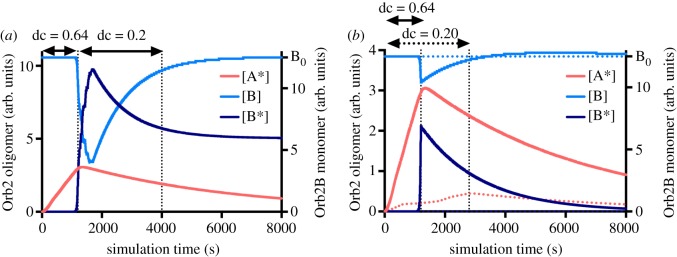
Consequences of the summation property of the Orb2 model. (*a*) The combination of a relatively short and strong stimulation (*ν* = 0.15 Hz, d.c. = 0.64, Δ*t* = 1200 s), with a consolidating low-frequency stimulation (*ν* = 0.15 Hz, d.c. = 0.20, Δ*t* = 2800 s) induces the long-term aggregation of Orb2, while (*b*) each single stimulation (solid/dotted lines) does not.

In order to dissect the individual role of the period and of the duty cycle in the pulse wave stimulation, we ran multiple simulations of the model where the period of the stimulation pulse was set constant, while changing the duty cycle (figure 2*c*, *ν* = 0.15 Hz), and vice versa (figure 2*d*, *dc* = 0.5). The simulations show that the system, as we modelled it, is more sensitive to the mean effective stimulation time rather than to the frequency of stimulation and that the responsiveness of the aggregation to a given duty cycle depends on the total time of stimulation. That is, if we consider again figure 2*c*, a rhythmic stimulation with a duty cycle of 0.4 which ceases after 4000 s does not trigger the self-sustaining aggregation of Orb2B, but a prolonged stimulation with that same duty cycle could induce the oligomerization. The intrinsic summation property and the synaptic activity gating of the model also imply the possibility that a relatively strong and brief stimulation, if followed by a consolidating low-frequency stimulation (see Material and methods), could also induce Orb2 aggregation (figure 3). As will be discussed below, the stimulation parameters (in particular, the use of a frequency range <1 Hz) were chosen in order to mimic the data from Orb2 *in vitro* aggregation kinetics [25] and the dopaminergic stimulation patters which have been associated with the medium- to long-term memory transition in *Drosophila* [29].

**Figure 4.**
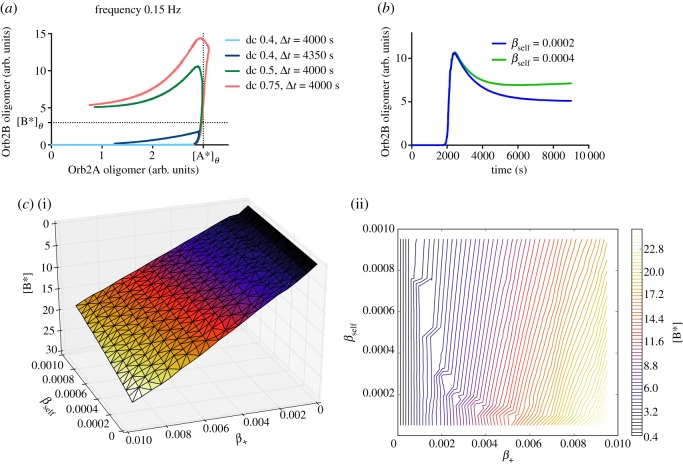
Role of different parameters of the model in the final aggregation outcome. (*a*) The presence of steep sigmoidal functions implies that the formation of a stable Orb2B aggregate depends on reaching the threshold size for the Orb2A aggregate [A^*^]_*theta;*_ and the self-templating threshold size for the Orb2B aggregate [B^*^]_*theta;*_. (*b*) The size of the mature Orb2 aggregate at the steady state depends on the ability of the aggregate to self-template new monomers, which in the model is described by *β*_self_. (*c*) Relationship between the size of the steady-state Orb2B aggregate ([B^*^]) and the dynamics of synaptic Orb2B, in particular, related to the parameters *β*_+_ and *β*_self_, visualized with a surface (*c*(i)) or a contour plot (*c*(ii)).

It can be easily recognized that the main driving force of this behaviour is that the levels of [A^*^] and [B^*^] have to reach the ‘oligomer’ thresholds [A^*^]_*theta;*_ and [B^*^]_*theta;*_ in order to trigger both the heteromeric nucleation and the self-templating state (see figure 4*a*). As a consequence of this, we can predict that the aggregation propensity of Orb2A—in particular, the ratio between *α*_agg_ and *α*_ex_—might control the length of stimulation required to induce LTM. For example, a mutated variant of Orb2A with delayed, but not abolished, aggregation would require a stronger stimulation to establish the memory. The shape of the functions describing [A^*^] and [B^*^] reflects the assumptions underlying the model. [B] starts aggregating when [A^*^] reaches a threshold level [A^*^]_*theta;*_. When [B^*^] grows up to a second threshold [B^*^]_*theta;*_, it inhibits the production of [A] and the aggregation seed [A^*^] starts disappearing. When the level of [A^*^] falls below the aforementioned threshold, the synaptic activity-dependent component of [B^*^] formation goes to zero. At this point, [B^*^] level becomes governed by its self-templating propensity: some values of *β*_self_ and *β*_ex_ would determine the monotonic decrease of [B^*^] seen in figure 3, while others would determine its monotonic increase to a different steady-state size of the aggregate (see figure 4*b*,*c*). Also, we have investigated whether there is a dependency between the steady-state levels of aggregated Orb2B and the baseline synaptic levels of monomeric Orb2B—that we have modelled to be *β*_+_/*β*_d_ units. In other words, we asked whether there are values of [B]_0_ where the availability of Orb2B monomers becomes a limiting factor in the aggregation model. Figure 4*c* shows that this can be the case and that the dependency can be partially compensated by assuming a higher self-seeding propensity of the Orb2 aggregate.

### 2.3. Protein aggregation-dependent plasticity and synaptic facilitation

Amyloid-like aggregation of Orb2 is directly linked to long-term memory in *Drosophila*: this implies the possibility to integrate the model in synaptic rules that include proteins regulated by Orb2. For example, there is evidence that Orb2 aggregation directly induces translation of the kinase CamKII, a fundamental component in various synaptic plasticity rules [30,31], and that the Orb2 mammalian homologue CPEB3 regulates activity-dependent translation of the AMPAR subunit GluA1 [17] and thus influences the local Na^+^ permeability.

One could thus argue that, if a salient condition has been presented for enough time to trigger the formation of an amyloid aggregate, after a time of latency *τ* between the aggregation and the consequent translational activation of pro-plasticity factors [32,33], the synapse transitions to a plasticity-prone resting state. For example, the variation of the local levels of CamKII in the resting-state synapse can be modelled as:

2.1 $$\frac{{\left[\mathrm{CamKII}\right]}^{\;\mathrm{post}}}{{\left[\mathrm{CamKII}\right]}^{\;\mathrm{pre}}}=\rho \cdot [B^{*}](t-\tau )$$

where *ρ* is a parameter that can be derived experimentally, for instance, using fluorescent reporters flanked by the UTR regions of the protein under study [23].

Moreover, a PADP criterion could be easily extended to take into account the multiple parallel processes of LTM, such as structural synaptic growth and molecular fingerprint modification, which depend on local translation.

## 3. Discussion

We presented a model of continuous differential equations for Orb2-aggregation and mechanisms of synaptic plasticity it may mediate. This synaptic plasticity mechanism has been shown to be important for a paradigm of LTM in the fly, but the interest of aggregation-dependent synaptic plasticity goes beyond this specific example and could represent a more general mechanism. The simulations performed show that the model is quite consistent with the molecular behaviour of Orb2 aggregates seen *in vitro* and *in vivo*. Orb2 protein aggregation is likely to be a detector of salient (i.e. biologically important) activity and a self-sustaining and self-limiting memory device whose outcome is very reliable and stable. Moreover, the assumption of a self-templating property of the Orb2B aggregate makes it possible to argue that a targeted synaptic activity is not critical for the maintenance of memory after LTM formation.

Another focal point in our model is the behaviour of the aggregation according to the stimulation pattern. Using a binary periodic stimulation, we have shown that our system detects the likelihood of stimulation in a given time window, rather than the frequency of the stimulation *per se*. Experiments in *Drosophila* have revealed that hours-long rhythmic dopaminergic stimulation could gate the transition between ARM and LTM [29]. If our assumptions are correct, the model would predict that a functionally equivalent stimulation, if associated with Orb2-mediated memory phenomena, should be permissive for the transition to LTM and the frequency of the stimulation would not be instructive in this process. Also, it would be indirect evidence of an Orb2 role in tuning the molecular pathways at the synapse from mid-lasting memory (ARM) to long-term memory (LTM). In this scenario, the insurgence of rhythmic (or resonating) stimulation patterns would be involved in reaching a sufficient level of mean activity during the Orb2 aggregation time window, which could induce the Orb2b aggregation in specifically Orb2A-tagged synapses. From an experimental point of view, this hypothesis could be assessed through, for example, optogenetic control of specific populations of dopaminergic neurons in the mushroom body. Also, our model predicts that the Orb2 physiological aggregates at the synaptic level function as a high-pass filter which would discriminate the stronger, experience-evoked rhythmic stimulation from the spurious ones. Moreover, the self-templating property of the mature Orb2 aggregate—which has been consistently shown in the Orb2 homologue ApCPEB—implies that once the aggregate is formed, no further synaptic recall would be required for the maintenance of the LTM. A further characteristic which emerged from the modelling is that the seeded aggregation process has an intrinsic ability to integrate the synaptic activity through time and locally. If this property was experimentally confirmed, the Orb2A-Orb2B system would extend the ability of synapses to integrate information from the classic spatial cooperativity with an integration time of a few milliseconds (as in STDP) to a more complex spatio-temporal pattern. However, it must be pointed out that some kinds of LTM are established after a short training period, such as in the Associative Olfactory-Reward memory, which has been shown to require the Orb2 aggregation for memory maintenance [15]. The current choice of σ(*t*) fails to extend our model to such phenomena. It is possible that the paradox of a quickly-inducible LTM with a stable molecular effector that is likely to build up in a few hours could be solved by taking into account a cascade of transient secondary mediators. For example, the synaptic levels of the Orb2A interaction partner LimK are actively regulated according to the synapse activity [21]. On the other hand, it could also be reasonable to assume that any LTM training, be it a 2 h Male Suppression Courtship protocol [10,15,18] or a 2 min olfactory reward one, would elicit a low-frequency recurrent synaptic activity pattern in a specific group of cells [29].

However, it is necessary to point out some limitations arising from such a model, because of the simplifying assumption we had to choose. Being a continuous model and considering the two states of the protein (free and aggregated) as two ‘compartments’, the model does not take into account the probabilistic aspects of the local aggregation (for example, the sensitivity to the fluctuations of protein concentration). Fluctuations in protein concentration might be particularly relevant, for the small volume and low protein numbers in a dendritic spine. The relationship between fluctuation of the levels of active protein species, their kinetic parameters and memory was shown theoretically and experimentally for PKA and ERK pathways in *Aplysia* [34]. Also, the model does not describe the chemical kinetic aspects such as aggregate polarization, the general mechanism of aggregation and so on. Given the little knowledge of molecular interacting partners of the aggregate and the pathways of cellular regulation of Orb2 aggregates [20,21,35,36], and the lack of a detailed quantitative study of Orb2A–Orb2B homo- and hetero-induced aggregation, we found it more conservative to model the overall known phenotype of Orb2 aggregate rather than to guess the molecular-level behaviour of the involved proteins. Kinetic datasets obtained through standardized methods would be required in order to write a master equation for Orb2 that would take into account the molecular steps of the aggregation, as has been done with more studied amyloidogenic proteins such as the A*β*_42_ peptide [37,38]. Other aspects of the model, now set as constant or null, for the sake of simplicity, that will have to be considered in future implementations of the model are: the transition from a point synapse to a spatially extended one and a more complex dynamic of Orb2A and Orb2B mRNAs and proteins, including diffusive components, RNA degradation, local depletion upon translation and/or aggregation and so on. It would be significant to investigate a differential diffusivity between Orb2A or Orb2B free proteins and Orb2 aggregate—which *in vivo* probably characterizes the local (synaptic) specificity of PADP.

Prion aggregation has been long recognized as a powerful mechanism of molecular memory related to LTM [26,39] and models of synaptic plasticity exist that include a step of protein translation [40]. To the best of our knowledge, with the present model, we performed the first attempt to take into account both protein aggregation in a physiological context as well as the local translation during synaptic facilitation processes. The paradigm of physiological prion aggregation requires tight feedback mechanisms for the species involved in order to avoid the uncontrolled formation of amyloids, which is potentially harmful to the neuron. This approach could be further applied to other synaptic plasticity phenomena which include local translation, such as the BDNF-induced plasticity [41] or mTORC-dependent plasticity [42]. There are other phenomena which are mediated by the formation of a stably active biological entity, just like the Orb2 functional amyloid, and are involved in memory induction and/or maintenance. A paradigmatic case, albeit controversial, is the kinase PKMζ, which is thought to be involved in long-term memory maintenance [43]. PKMζ is locally translated after a salient synaptic activity and is constitutively active (it does not rely on a secondary messenger to maintain its activity), in a way which is similar to the Orb2 aggregates here presented. The model could be easily used as a starting point for a generalization of PADP in a plasticity rule which depends on synaptic protein network modification through local translation.

## 4. Material and methods

### 4.1. Equations of the model

The local elevation of Orb2A levels depends on synaptic activity [21], particularly mediated by the dopamine receptor DR1 [23]. In order to model the effect of dopaminergic activity on the system, we consider each dopamine receptor to assume a state 1 or 0 at a given time, and the synaptic activity to be the average activation of the receptor population

4.1 $$\sigma (t)=s(t)\cdot \frac{\sum_{i}D1_{i}(t)}{N}=s(t)\cdot \langle D1\rangle ,$$

where *D*1_1_, *D*1_2_, … , *D*1_*N*_ is the sequence of the states of the dopamine receptors at the synapse at a given time, and *s*(*t*) takes into account every other non-DR1 synaptic contribution (e.g. ante- or retrograde action potentials, other neuromodulator signalling and so on).

The Orb2A accumulation is regulated by the stabilizing effect of Tob/LimK-mediated phosphorylation of Orb2A and by the destabilization of phosphorylated Orb2A interacting protein Tob [21]: the superposition of these processes likely identifies a time window for the synaptic oligomerization of Orb2A. Also, if Orb2A is necessary for the acquisition of memory and not during its long-term maintenance [23], it has to be assumed that the formation of the Orb2 final aggregate would in some way inhibit the late accumulation of Orb2A in order to avoid an uncontrolled prionic aggregation. The equation (4.2) tries to include the described properties of Orb2A accumulation:

4.2 $$\frac{\text{d}[A]}{\text{d}t}=\alpha_{\mathrm{acc}}\sigma (t)\Theta_{B^{*}}^{1\to 0}-\alpha_{\deg}[A],$$

where *α*_acc_ is the local accumulation rate of Orb2A, which can be assumed to be due to the increase of the stability of Orb2A which is induced by the synaptic activity, *α*_deg_ is the local depletion rate of the Orb2A species and $\Theta_{B^{*}}^{1\to 0}(B^{*})$ is a continuous sigmoid function which assumes values close to zero when the amount of Orb2 aggregate (see equation (4.6)) approaches a threshold value ${\left[B\right]}_{\theta ,A}^{*}$ and close to one for smaller values.

Orb2A has a strong intrinsic tendency to aggregation [15], so we define its aggregation as a simple first-order equilibrium reaction:

4.3 $$\frac{\text{d}[A^{*}]}{\text{d}t}=\alpha_{\mathrm{agg}}[A]-\alpha_{\mathrm{ex}}[A^{*}]$$

where *α*_agg_ is the aggregation rate and *α*_ex_ is the aggregate exit rate, which takes into account the fact that the Orb2A aggregation seed does not form after every synaptic stimulation. Applying the conservation of Orb2A species to equations (4.2) and (4.3), we obtain:

4.4 $$\frac{\text{d}[A]}{\text{d}t}=\alpha_{\mathrm{acc}}\sigma (t)\Theta_{B^{*}}^{1\to 0}+\alpha_{\mathrm{ex}}[A^{*}]-(\alpha_{\deg}+\alpha_{\mathrm{agg}})[A]$$

The Orb2A/Orb2B hetero-oligomerization, which is likely to constitute the mature Orb2 aggregates, depends on both the presence of an Orb2A aggregation seed [15,18] and of synaptic stimulation [23]. It has been shown, at least for *Aplysia* CPEB amyloid-like aggregates, that the dimensions of the higher-order aggregates (also called *puncta*) are kept at a steady level [13], with a turnover rate of about 20% in 48h. This steady-state behaviour could also be applied to Orb2. Also, the Orb2 aggregate self-sustaining steady state appears to be independent of the presence of Orb2A after reaching the mature state. In order to model this complex behaviour, we consider a simplification: both the mRNA and the protein of Orb2B are present at high levels in the cytoplasm, so we assume that the Orb2B protein is abundant at the dendrite level, because the local levels of the protein are buffered against a much larger somatic reservoir:

4.5 $$\frac{\text{d}[B]}{\text{d}t}=\beta_{+}-\beta_{d}[B],$$

where *β*_+_ represents all the processes that could enrich the local population of monomeric Orb2B in the synapse (such as the transport from the dendritic shaft/axon), and *β*_d_ is the local depletion rate of the Orb2B monomer. We then propose that the Orb2B aggregation presents two components: an Orb2A oligomer- and synaptic activity-mediated aggregation [15,23], and a self-sustaining aggregation [13,15]. The equation for Orb2B aggregation can be expressed as follows:

4.6 $$\frac{\text{d}[B^{*}]}{\text{d}t}=(\beta_{\mathrm{agg}}\Theta_{A^{*}}^{0\to 1}\sigma (t)+\beta_{\mathrm{self}}\Theta_{B^{*}}^{0\to 1})[B]-\beta_{\mathrm{ex}}[B^{*}]$$

where [B] and [B^*^] are the amount of Orb2B in monomer and in aggregated state, *β*_agg_ is the Orb2A seed-dependent aggregation rate, *β*_self_ is the Orb2B aggregate-dependent aggregation rate, and *β*_ex_ rules the aggregate dissociation. For the sake of simplicity, we assume that the link between synaptic activity and the Orb2B hetero-induced aggregation has the same shape as the one that is involved in Orb2A oligomerization. Again, $\Theta_{B^{*}}^{0\to 1}(B^{*})$ is a continuous sigmoid function which assumes values close to one when the amount of Orb2 aggregate approaches a threshold value ${\left[B\right]}_{\theta }^{*}$ and close to zero for smaller values: that is, the Orb2B aggregate must have a certain size in order to self-induce the aggregation of new monomers. For the sake of simplicity, the equation (4.6) does not take into account the possible weak homoinduced Orb2B aggregation, which has been hypothesized in order to explain the residual LTM observed in Drosophila strains where Orb2A is aggregation-defective [18], as it is unlikely that this mechanism would affect significantly the dynamics of physiological Orb2 aggregation. It is also worth noticing that in equation (4.6), we assume that the self-induced aggregation does not require synaptic activity. The equation that describes the Orb2B monomer levels at the synapse is thus:

4.7 $$\frac{\text{d}[B]}{\text{d}t}=\beta_{+}+\beta_{\mathrm{ex}}\;[B^{*}]-(\beta_{d}+\beta_{\mathrm{agg}}\Theta_{A^{*}}^{0\to 1}\sigma (t)+\beta_{\mathrm{self}}\Theta_{B^{*}}^{0\to 1})\;[B].$$

### 4.2. Numerical simulations

Numerical simulations of equations (4.3), (4.4), (4.6) and (2.1) were performed using a custom-made script of the model (electronic supplementary material, file S1 online) with parameters as shown in table 1. The parameters were chosen after repeated evaluation of the model through computer simulations, so that they would maintain biologically compatible ratios and a dynamic grossly coherent with the experimental data. The sigmoid functions $\Theta_{B^{*}}^{1\to 0}$, $\Theta_{A^{*}}^{0\to 1}$ and $\Theta_{B^{*}}^{0\to 1}$ present in equations (4.4), (4.6) and (4.7) have been approximated with the steep logistic functions

4.8 $$\Theta_{X^{*}}^{0↔1}([X^{*}])={\left(1+\text{exp}(\pm 50([X^{*}]-{\left[X^{*}\right]}_{\theta }))\right)}^{-1/2}.$$

**Figure 5.**
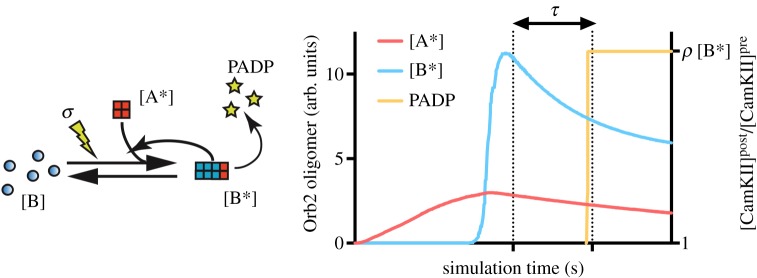
PADP and synaptic plasticity. Representative simulations of equations (4.4), (4.6) and the PADP model (equation (2.1)).

For the sake of simplicity, in the simulations shown in figures 2 and 5 we set the system so that the whole receptor population located in the synapse (see equation (4.1)) would activate at the same time in the presence of dopamine and set *s*(*t*) = 1, so the stimulation function σ(*t*) can be approximated with a periodic pulse (i.e. rectangular) wave

4.9 $$\sigma (t)=s(t)\cdot \langle D1\rangle {|}_{t}∼\text{rect}(t),$$

whose characterizing parameters are thus the wave *frequency* (*ν*) and its *duty cycle* (*dc*)

4.10 $$\mathrm{rect}(t)=\left\{ \begin{array}{cc} +1 & \text{k}\nu^{-1}\leq t<dc+\text{k}\nu^{-1}\\ 0 & dc+\text{k}\nu^{-1}\leq t<\text{k}\nu^{-1} \end{array}\right.$$

where k is a natural number. For the simulation shown in figure 3, a more complex pattern of stimulation was chosen (see electronic supplementary material, file S2):

4.11 $$\sigma (t)=s(t)\cdot \langle D1\rangle {|}_{t}=\left\{ \begin{array}{ll} \text{rect}\;(\nu =0.15\;\text{Hz},\;dc=0.64) & \text{if}\;\;t\leq 1200\;\text{s}\;\\ \text{rect}\;(\nu =0.15\;\text{Hz},\;dc=0.20) & \text{if}\;\;1200<t\leq 4000\;\text{s}\\ 0 & \text{if}\;\;t>4000\;\text{s} \end{array}\right.$$

that, ideally, represents the succession of a strong, middle-term biologically relevant activity and a rhythmic low-frequency consolidating synaptic activity, which by themselves would not induce the simulated aggregation of Orb2 in the given time window.

**Table 1.** Parameters of the Orb2 model described in the main text and used in the numerical simulations.

parameter	value	parameter	value	parameter	value
*α*_acc_	0.005 units Hz	*α*_deg_	0.002 Hz	*α*_agg_	0.008 Hz
*α*_ex_	0.001 Hz	[A^*^]_*θ*_	3 units		
*β*_+_	0.005 units Hz	*β*_d_	0.0004 Hz	*β*_agg_	0.05 Hz
*β*_ex_	0.0005 Hz	*β*_self_	0.0002 Hz		
[*B*]_0_	*β*_+_/*β*_d_ units	[B^*^]_*theta;*_	3 units	〈*D*1〉	1

In order to prove the dependency of the aggregation of Orb2B on the values of [B]_0_ and *β*_self_ we performed multiple evaluations of the steady-state value of [B^*^] while changing the parameters *β*_self_ and *β*_+_ so to span three different orders of magnitude (see electronic supplementary material, file S3 online).

## Supplementary Material

Supplementary File 1

## Supplementary Material

Supplementary File 2

## Supplementary Material

Supplementary File 3

## Data accessibility

All python scripts used to generate the data are available on the Dryad Digital Repository [44].
